# Supplementary material for: Tandem association of CLOCK:BMAL1 complexes on DNA enables recruitment of CBP/p300 through multivalent interactions
Source: bioRxiv. 2025 Sep 7:2025.09.04.674342. Preprint. [Version 1] doi: 10.1101/2025.09.04.674342 (PMC12424780; doi:10.1101/2025.09.04.674342)

Supplementary Materials

Tandem association of CLOCK:BMAL1 complexes on DNA enables recruitment of CBP/p300 through multivalent interactions

Diksha Sharma[1*], Lisa Stoos[2*], Megan R. Torgrimson[1*], Priya Crosby[1$], Kyle M. Franks[1], Nicole C. Parsley[1], Miles Membreno[1], Georg Kempf[2], Lukas Kater[2], Chelsea L. Gustafson[1], Hsiau-Wei Lee[1], Seth M. Rubin[1], Alicia K. Michael[2#], Nicolas H. Thomä[2,3], Carrie L. Partch[1,4,5]

[1] Department of Chemistry and Biochemistry, University of California Santa Cruz, Santa Cruz, CA USA
[2] Friedrich Miescher Institute for Biomedical Research, Basel, Switzerland
[3] Swiss Federal Technology Institute of Lausanne, Lausanne, Switzerland
[4] Center for Circadian Biology, University of California San Diego, La Jolla, CA USA
[5] Howard Hughes Medical Institute, University of California Santa Cruz, Santa Cruz, CA USA
*equal contributions

[$]Current address: Institute of Cell Biology, School of Biological Sciences, University of Edinburgh, United Kingdom
[#]Current address: Institute for Science and Technology Austria, Vienna, Austria

Correspondence to: cpartch@ucsc.edu

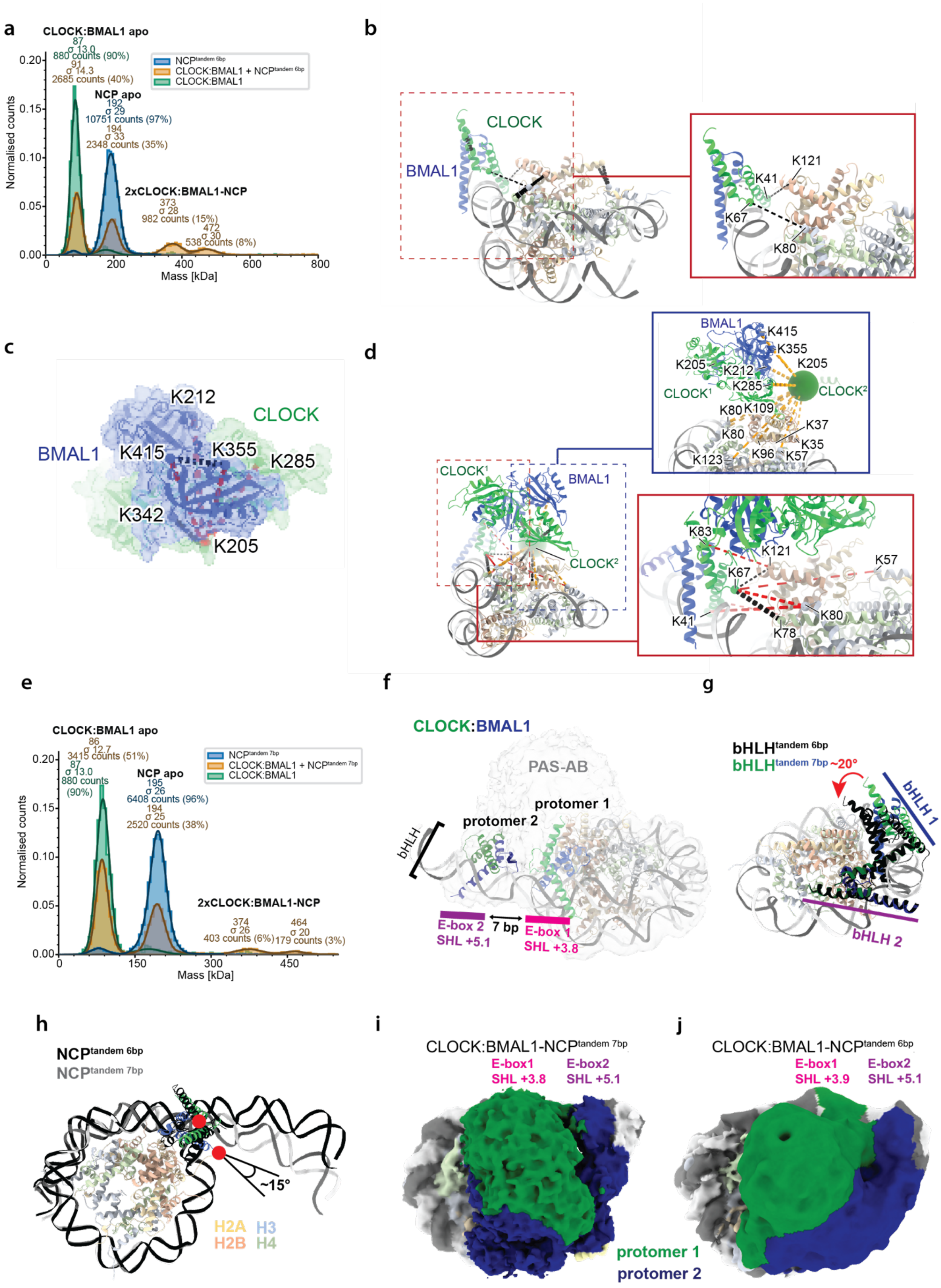

a
CLOCK:BMAL1 apo
87
σ 13.0
880 counts (90%)
91
σ 14.3
2685 counts (40%)
NCP apo
192
σ 29
10751 counts (97%)
194
σ 33
2348 counts (35%)
2xCLOCK:BMAL1-NCP
373
σ 28
982 counts (15%)
472
σ 30
538 counts (8%)
NCP tandem 6bp
CLOCK:BMAL1 + NCP tandem 6bp
CLOCK:BMAL1
Normalised counts
Mass [kDa]
b
CLOCK
BMAL1
K121
K41
K67
K80
c
K212
CLOCK
BMAL1
K415
K355
K285
K342
K205
d
BMAL1
K415
K355
K205
K212
K205
K285
CLOCK¹
CLOCK²
K80
K109
K37
K80
K35
K123
K96
K57
CLOCK¹
BMAL1
CLOCK²
K83
K121
K57
K67
K41
K80
K78
e
CLOCK:BMAL1 apo
86
σ 12.7
3415 counts (51%)
87
σ 13.0
880 counts
(90%)
NCP apo
195
σ 26
6408 counts (96%)
194
σ 25
2520 counts (38%)
2xCLOCK:BMAL1-NCP
374
σ 26
403 counts (6%)
464
σ 20
179 counts (3%)
NCP tandem 7bp
CLOCK:BMAL1 + NCP tandem 7bp
CLOCK:BMAL1
Normalised counts
Mass [kDa]
f
CLOCK:BMAL1
PAS-AB
protomer 1
protomer 2
bHLH
E-box 2
SHL +5.1
7 bp
E-box 1
SHL +3.8
g
bHLH tandem 6bp
bHLH tandem 7bp
~20°
bHLH 1
bHLH 2
h
NCP tandem 6bp
NCP tandem 7bp
~15°
H2A
H3
H2B
H4
i
CLOCK:BMAL1-NCP tandem 7bp
E-box1
SHL +3.8
E-box2
SHL +5.1
protomer 1
protomer 2
j
CLOCK:BMAL1-NCP tandem 6bp
E-box1
SHL +3.9
E-box2
SHL +5.1

Figure S1: Characterization of CLOCK:BMAL1-NCP complexes. a) Molecular mass distribution histogram of CLOCK:BMAL1-NCP$^{tandem\ 6bp}$. CLOCK:BMAL1 and the nucleosomes were first measured individually at 20 nM and in a 1:6 ratio. CLOCK:BMAL1 primarily forms complexes with nucleosomes in a 1:2 complex with a minority species of 1:3 stoichiometry. b) Cross-links between the bHLH domain of CLOCK:BMAL1 and histones allow the specific identification of CLOCK and BMAL1. c) Cross-links mapped to the PAS domains of a single CLOCK:BMAL1 heterodimer. Sterically incompatible cross-links are depicted in red. d) Tentative model for the first CLOCK:BMAL1 protomer. CLOCK K205 from the second protomer, which makes multiple cross-links is depicted as a sphere (blue box). Cross-links did not allow unambiguous model building and suggest that multiple conformations for the PAS domains of the second protomer are possible. Putative inter-CLOCK:BMAL1 cross-links would be sterically incompatible when mapped to a single heterodimer (see panel c). e) Molecular mass distribution histogram of CLOCK:BMAL1-NCP$^{tandem\ 7bp}$. CLOCK:BMAL1 and the nucleosomes were first measured individually at 20 nM and in a 1:6 ratio. CLOCK:BMAL1 primarily forms complexes with nucleosomes in a 1:2 complex with a minority species of 1:3 stoichiometry. f) Map-model overlay for CLOCK:BMAL1-NCP$^{tandem\ 7bp}$. The resolution of the map only allowed confidently building of the bHLH domains of both protomers. g) Comparison of the two models, aligned on the second bHLH domain at SHL+5.1 indicates a rotation of ~ 20º of the first bHLH domain. h) Comparison of the DNA trajectory between CLOCK:BMAL1-NCP$^{tandem\ 6bp}$ and CLOCK:BMAL1-NCP$^{tandem\ 7bp}$. Only the internal bHLH domain is depicted for clarity. i-j) Top view of the cryo-EM map obtained for CLOCK:BMAL1 binding to tandem E-boxes with 7 bp spacing (i) or 6 bp spacing (j).

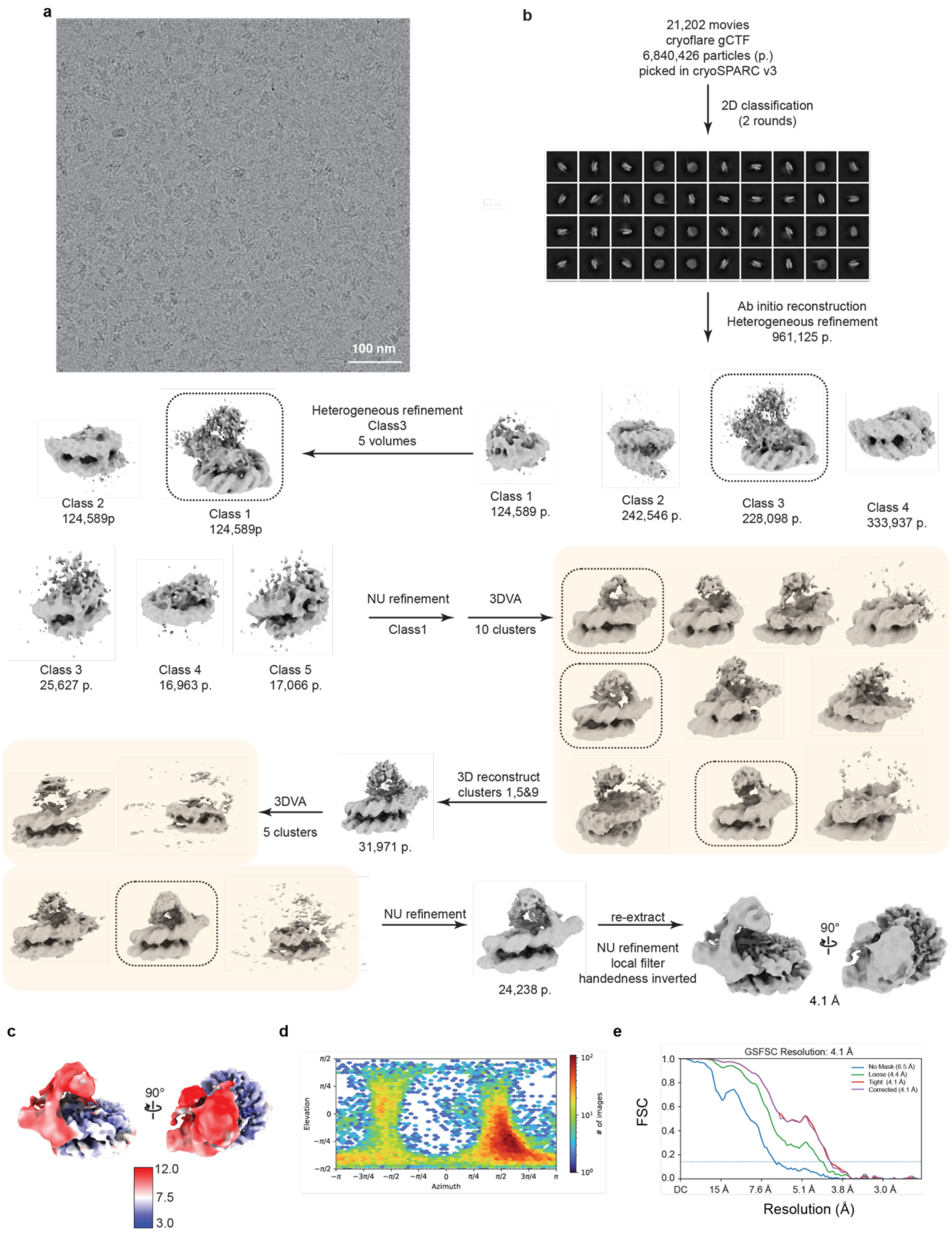

Figure S2: cryo-EM data processing workflow for CLOCK:BMAL1-NCP$^{tandem\ 6bp}$. a) Representative cryo-EM micrograph. b) See methods. The movies were pre-processed within cryoFLARE gCFT and the resulting micrographs were imported in cryoSPARC v3 for further processing. Multiple rounds of classification, refinement and 3D variability analysis followed by non-uniform refinement (NU refinement) resulted in the final map. The dashed boxes indicate the maps and sets of particles used for the following step in

the data processing workflow. c) Local resolution map for the 4.1 Å final map in b. d) Angular distribution for the particles leading to the 4.1 Å resolution map. e) Gold-standard Fourier shell correlation (FSC) curve for the final map.

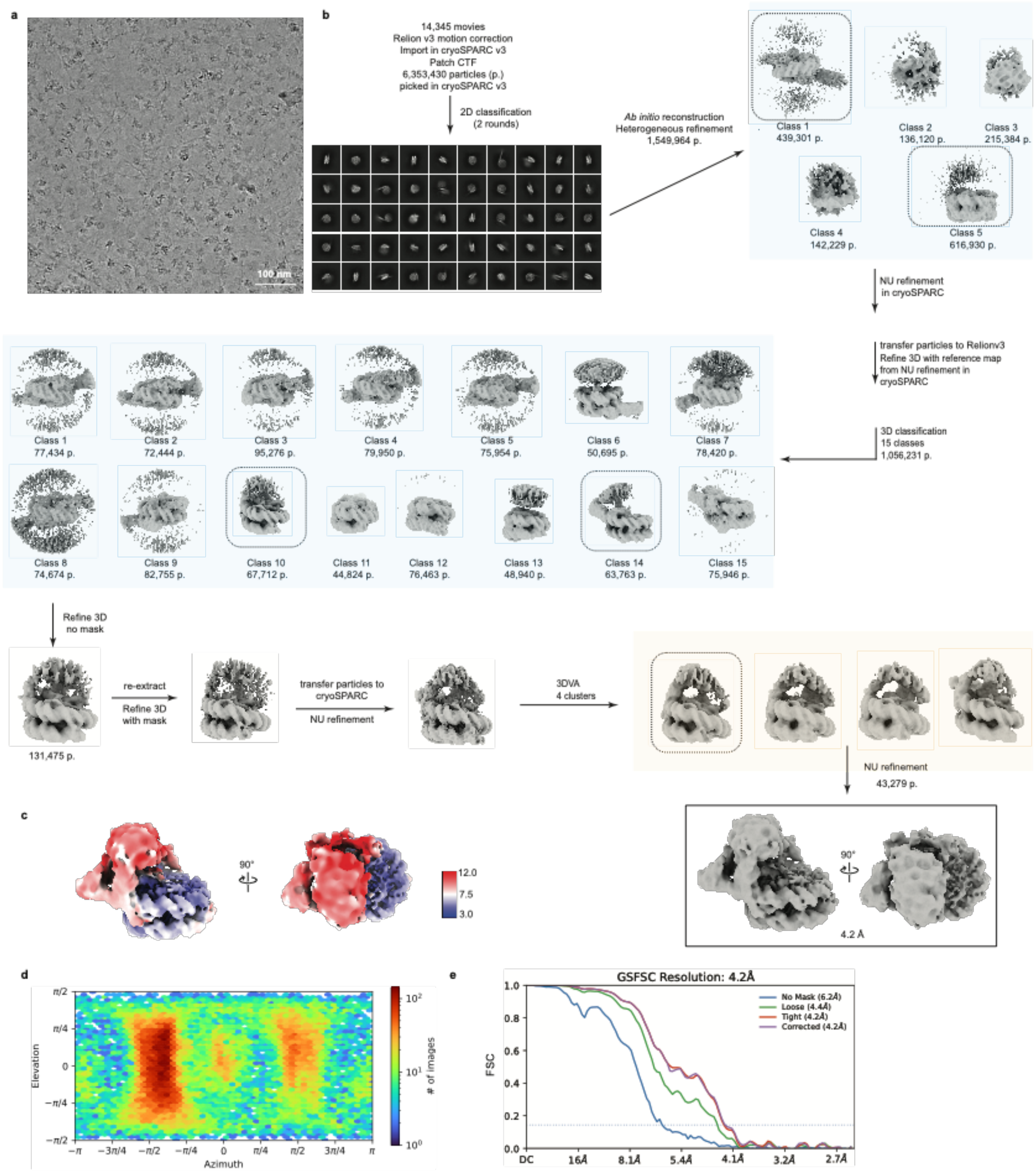

Figure S3: cryo-EM data processing workflow for CLOCK:BMAL1-NCP$^{\text{tandem 7bp}}$. a) Representative denoised cryo-EM micrograph. b) See methods. The movies were pre-processed within cryoFLARE. The resulting micrographs were imported in cryoSPARC v3 for particle picking. After multiple rounds of classification and refinements, the remaining particles were imported into Relion v3 for further classification and refinements. The final refinements and 3D variability analysis were performed in cryoSPARC v3. The final map is highlighted by a black box. The boxes defined by the dashed lines indicate the good maps and particle sets used for the following step in the data processing

workflow. c) Local resolution map for the 4.2 Å final map in b. d) Angular distribution for the particles leading to the 4.2 Å resolution map. e) Gold-standard Fourier shell correlation (FSC) curve for the final map.

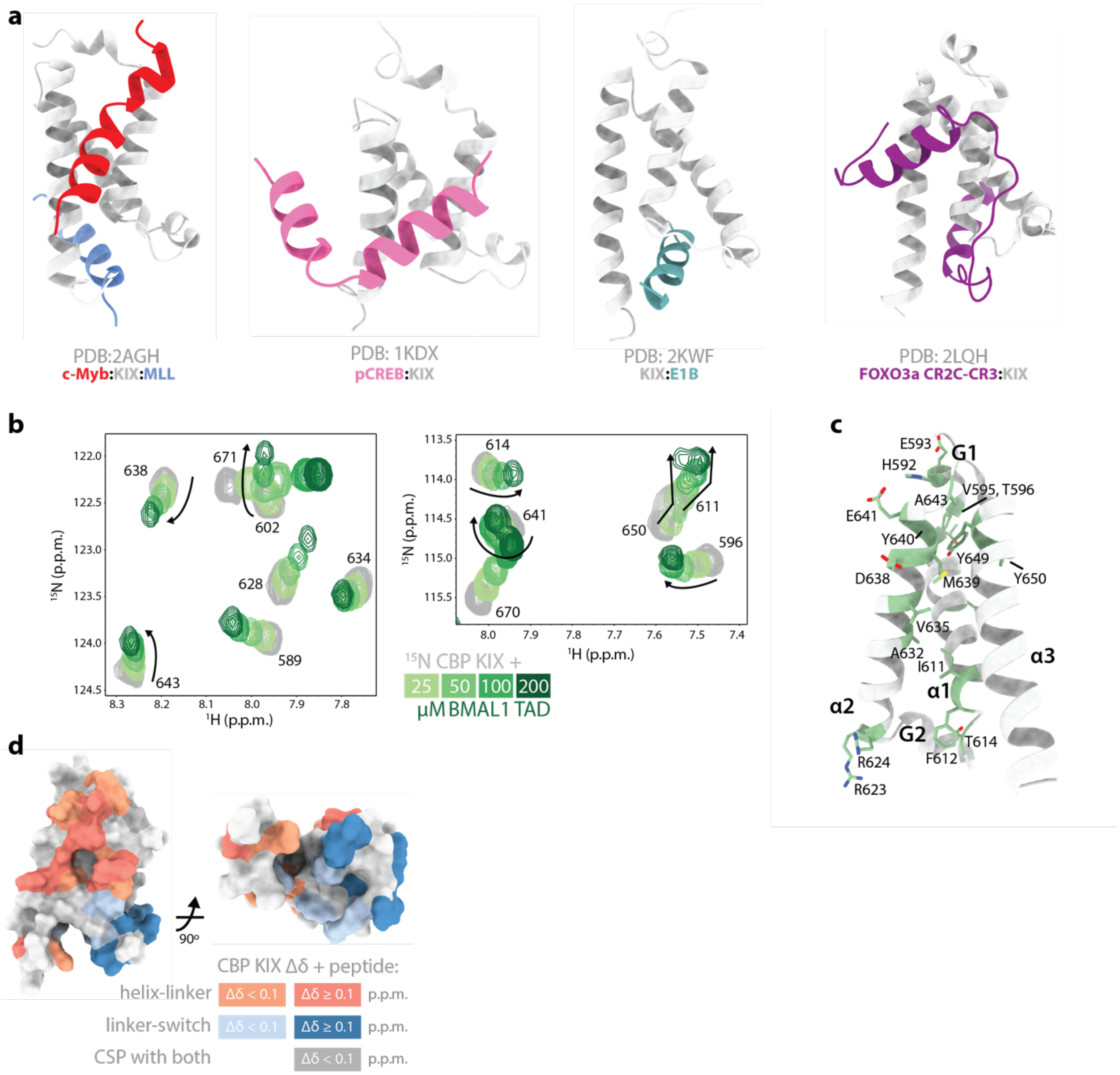

Figure S4: Selected peaks in CBP KIX with curved CSP trajectories. a) Structures of CBP KIX complexes with different transcription factors. b) Selected regions from the ^{15}N-^{1}H HSQC of 100 µM ^{15}N CBP KIX titrated with the BMAL1 TAD as indicated (from full spectrum shown in Fig. 2b). Chemical shifts with curved trajectories are marked with arrows. c) CBP KIX domain (PDB 2AGH, light gray) with secondary structure annotated and residues exhibiting curved chemical shift trajectories colored in green. For reference, the MLL1 binding site is located at the bottom of the KIX domain between the alpha-2 and alpha-3 helices beneath the G2 helix. d) CSPs of ^{15}N CBP KIX bound to BMAL1 TAD peptides helix-linker (salmon hues) or linker-switch (steel blue hues) from Fig. 2e mapped onto PDB 2AGH. Residues that shift with both peptides are shown in dark gray. See Table 2 for more information.

a

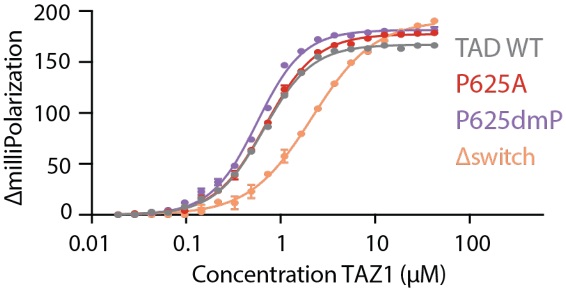

b

	Kd (µM)	Hill coeff.
WT	0.75 ± 0.14	1.59 ± 0.16
P625A	0.75 ± 0.05	1.55 ± 0.13
P625dmP	0.54 ± 0.12	1.71 ± 0.21
Δswitch	2.39 ± 0.23	1.12 ± 0.17

Figure S5: CBP TAZ1 binding to variants of the BMAL1 TAD. a) Fluorescence polarization data of CBP TAZ1 binding to different [5,6] TAMRA-BMAL1 minimal TAD probes as indicated: TAD WT (60% *trans,* 40% *cis*), P625A (all *trans)*, P625dmP (all *cis)*, Δswitch (truncated at F619Y) n = 3, mean ± s.d. b) K_D and Hill coefficients from FP binding data.

Table 1. Cryo-EM data collection, refinement and validation statistics

	Tandem 7bp<br>EMD-54869<br>PDB 9SFZ	Tandem 6bp<br>EMD-54868<br>PDB 9SFY
**Data collection and processing**		
Detector	Falcon4i	Falcon 4i
Magnification voltage (kV)	200	300
Electron exposure ($e^{-}/Å^2$)	50	50
Defocus range (μm)	-0.8 - -2	-0.8 - -2
Pixel size (Å)	0.84	0.845
Symmetry imposed	C1	C1
Initial particle images (no.)	6,353,430	6,840,426
Final particle images (no.)	43,279	24,238
Map resolution (Å)	4.2	4.1
FSC threshold	0.143	0.143
Map resolution range (Å)	3-12	3-12
**Refinement**		
Initial models used (PDB codes)	6T93, 4F3L, 4H10, 8OSL	6T93, 4F3L, 4H10, 8OSL
Model resolution (Å)		
FSC threshold	4.2	4.1
Map sharpening *B* factor ($Å^2$)	N.A	N.A
Model composition		
Non-hydrogen atoms	13467	12309
Protein residues	960	861
Nucleotides	264	256
Ligands	0	0
B factors ($Å^2$)	193.80	144.61
Protein	179.69	128.88
DNA	214.81	165.77
R.m.s. deviations		
Bond lengths (Å)	0.007	0.007
Bond angles (°)	0.867	0.931
Validation		
MolProbity score	0.76	1.06
Clashscore	0.85	2.61
Poor rotamers (%)	0.16	0.95
Ramachandran plot		
Favored (%)	99.04	97.98
Allowed (%)	0.96	1.90
Disallowed (%)	0.00	0.12
**Model-to-data fit**		
CCmask	0.6571	0.6062
CCbox	0.7680	0.7276
CCpeaks	0.6071	0.5550
CCvolume	0.6474	0.5986

Table 2. CBP KIX residues with CSPs mapped onto PDB 2AGH (chain 1.1/B).

Figure panel	Condition	CBP KIX residue
Fig. 2c	KIX with BMAL1 TAD,<br>$\Delta\delta$ < 0.1 p.p.m. (light green)	596,598,599,602,603,606,611,612,<br>620,621,622,624,625,628,631,632,<br>633,645,650,653,654,657,658,661,<br>662,664,668,670,671
	KIX with BMAL1 TAD,<br>$\Delta\delta$ > 0.1 p.p.m. (dark green)	588,589,591,592,595,597,604,605,<br>610,614,616,626,627,629,634,635,<br>636,637,638,639,640,641,643,644,<br>646,649,652,655,672
Fig. S4c	KIX with BMAL1 TAD,<br>Curved CSP trajectories	595,596,602,611,612,614,623,624,<br>632,635,638,639,640,641,643,649,671
Fig. 2e<br>Fig. S4d	KIX with TAD helix-linker<br>$\Delta\delta$ < 0.1 p.p.m. (light salmon)	590,594,603,605,642,645,647,656,<br>657,660,663,669
	KIX with TAD helix-linker<br>$\Delta\delta$ > 0.1 p.p.m. (salmon)	602,606,608,646,648,650,654,658,670
	KIX with TAD linker-switch<br>$\Delta\delta$ < 0.1 p.p.m. (light blue)	609,612,614,624,630,631,634,641,664
	KIX with TAD linker-switch<br>$\Delta\delta$ > 0.01 p.p.m. (blue)	615,616,620,621,623,625,626,632,<br>635,636
	KIX with helix-linker/linker-switch<br>$\Delta\delta$ > 0.05 p.p.m. (dark gray)	592,595,596,641

Uncropped Blots

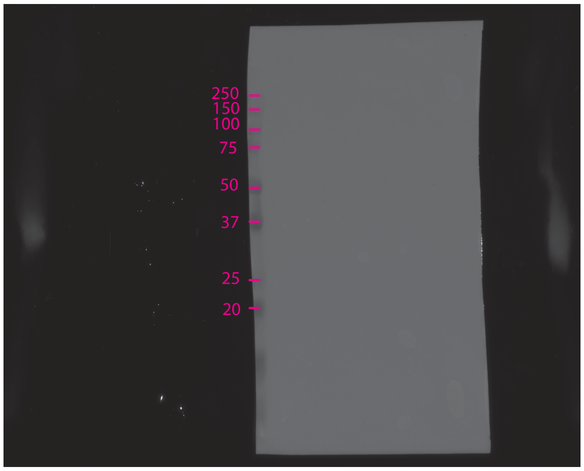

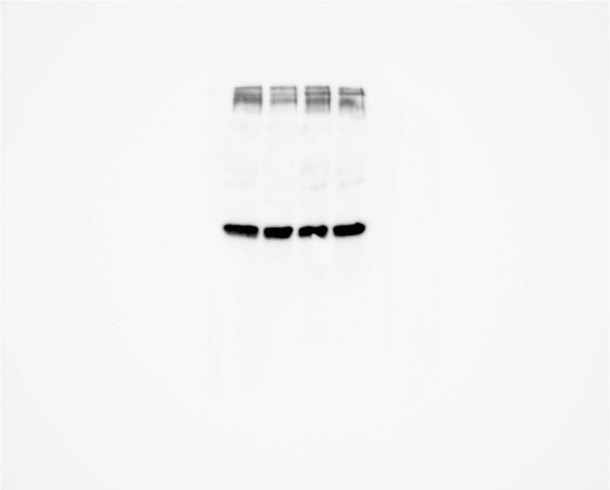

Supplement: Supplement 1 [file media-1.pdf]
